# Shengjing Capsule Improves Spermatogenesis through Upregulating Integrin *α*6/*β*1 in the NOA Rats

**DOI:** 10.1155/2019/8494567

**Published:** 2019-08-22

**Authors:** Jiamin Wang, Shankun Zhao, Lianmin Luo, Yangzhou Liu, Ermao Li, Zhiguo Zhu, Zhigang Zhao

**Affiliations:** ^1^Department of Urology & Andrology, Minimally Invasive Surgery Center, Guangdong Provincial Key Laboratory of Urology, The First Affiliated Hospital of Guangzhou Medical University, Guangzhou, Guangdong, China; ^2^Research Laboratory for Clinical & Translational Medicine, Medical School, University of South China, Hengyang 421001, China

## Abstract

**Objective:**

To evaluate the therapeutic effect of Shengjing capsules on nonobstructive azoospermia (NOA) in the rat model.

**Methods:**

Twenty-five male Sprague–Dawley rats were randomly divided into five groups as follows (*n*=5 per group): normal group, NOA group, and three Shengjing capsule treatment groups (low-dose, medium-dose, and high-dose groups, respectively). HE staining and semen smear were performed to assess sperm quality. The expression levels of PI3K/AKT and integrin *α*6/*β*1 were measured by qRT-PCR and western blot analyses.

**Results:**

In the NOA group, almost all of the seminiferous tubules were vacuolated with a thin layer of basal compartment containing some spermatogonial stem cells. The counts of sperms in the NOA group were strongly lower than those of the normal group (*P*=0.0001). The expression of PI3K/AKT and integrin *α*6/*β*1 was scarcely expressed in the NOA group. All indexes mentioned above were significantly different from those of the medium- and high-dose groups (*P*=0.001, all). The sperm count of rats treated with Shengjing capsules was significantly higher than that of the NOA group (*P*=0.0001). The rats of Shengjing capsule groups had more layers of spermatogonial stem cells and spermatocytes, and some had intracavitary sperms.

**Conclusions:**

Shengjing capsules may be a promising therapeutic medicine for NOA. The underlying mechanisms might involve activating SSCs by upregulating the integrin *α*6/*β*1 expression via the PI3K/AKT pathway.

## 1. Introduction

Infertility hampers about 15% of couples attempting pregnancy, and male factor infertility accounts for approximately half of all the reasons of this impaired fecundity [1]. The incidence of infertility is increasing year by year, and it has grown up to be a global health problem. Azoospermia, the medical condition that men do not have any measurable level of spermatozoa in their semen, is an extremely important contributor to male infertility. And nonobstructive azoospermia (NOA) is a more complicated infertility syndrome, with the azoospermia being secondary to a failure to produce sperm. NOA, commonly referred to as testicular failure, is classified into three subtypes on the basis of histopathological examination of testicular tissue, including hypospermatogenesis (HS), maturation arrest (MA), and Sertoli cell-only (SCO) syndrome [2]. Almost 10% of infertile men suffer from NOA. NOA is one of the most difficult conditions to treat. Microdissection testicular sperm extraction (micro-TESE) is the most common treatment for NOA [3]. However, 50–60% of the micro-TESE treatments failed to retrieve spermatozoa.

In recent years, many techniques, including spermatogonial stem cell (SSC) transplantation, testis tissue transplantation, and induced pluripotent stem cell and gene therapy, have emerged and developed. Intracytoplasmic sperm injection (ICSI) has also been improved, increasing the chances for men to father a child. However, the effective treatment for NOA patients is absent. Thus, it is urgently needed to find an efficient and feasible therapy.

NOA is characterized by severely impaired or nonexistent spermatogenesis [4]. This suggests that the improvement of NOA can be achieved by improving spermatogenesis. Spermatogenesis is a complex process that requires about 64 days in rats and 74 days in humans [5]. Sperm originates from SSCs, which are vitally crucial for spermatogenesis and male reproduction. SSCs serve as a basis for spermatogenesis throughout adult life by undergoing self-renewal and providing progeny cells that differentiate into spermatozoa [6, 7]. OCT4, MHC I, C-kit, *α*6-integrin, and *β*1-integrin are useful immunohistochemical staining markers of cell populations, which are highly expressed in stem cells. Especially, *α*6-integrin and *β*1-integrin were recognized as marker molecules on SSCs [8, 9].

The use of traditional Chinese herbal medicine to improve testicular spermatogenesis has been in China for more than 2,000 years. Shengjing capsules are made up of a variety of medicinal materials that improve sperm production, such as ginseng, *Cordyceps sinensis*, *Epimedium*, and medlar [10–14]. *Panax ginseng* extracts have shown significant improvements in sperm concentrations and motility in semen analysis from a randomized controlled trial [10]. Park et al. [11] reported that *Panax ginseng* plays an important role in improving sperm hyperactivation via the cation channel of the sperm protein gene expression. *Lycium barbarum* (*L. barbarum*), also named wolfberry, obviously has the protective effect on the spermatogenesis of rats with the impaired reproduction system induced by cyclophosphamide [12]. Icariin, the most metabolically active extract of *Epimedium*, has been proved to improve endothelial cell function in the penis and promote the formation of NO [13]. Additionally, *Cordyceps militaris* extracts significantly enhanced the sperm production at the end of the first month and peaked it at the second month [14]. Since its introduction in 2005, the Shengjing capsule is widely used clinically to improve male infertility due to poor semen quality [15]. Shengjing capsules also can improve spermatogenesis ability to improve oligozoospermia [16–18]. The safety of this medicine has been confirmed by clinical use. Shengjing capsules do not relieve obstruction in OA patients. In summary, Shengjing capsule can improve the quality of semen in patients with OA, although it can not relieve the obstruction. This requires more research with a higher level of evidence to support.

Shengjing capsules have the potential to improve NOA spermatogenesis, but their mechanism is not yet clear. We have for the first time explained that Shengjing capsules can upregulate the *α*6/*β*1 expression by the PI3K pathway to activate SSCs and improve the therapeutic effect of spermatogenic function on NOA rats.

## 2. Materials and Methods

### 2.1. Formula and Preparation

Shengjing capsule extracts, provided by Liao Yuan He Tang Pharmaceutical Co., Ltd. (Zunyi, China), were officially approved in the treatment of male infertility and asthenospermia by the State Food and Drug Administration of P.R. China (SFDA) (standard number WS-11457(ZD-1457)-2002-2011Z; national drug approval Z20027672). The Shengjing capsule manufacturer recommends 504 mg/day for pharmacological research in rats. Meanwhile, in the dose conversion between animals and humans, the US Food and Drug Administration guidance for industries [19] recommended the dose conversion coefficient of rats to be 6.2, and after conversion, the dosage for rats was close to 504 mg/day. Thus, based on its relative equivalent dose of 4.8 g/day in a 60 kg man, 504 mg/kg of the Shengjing capsule was selected in this study (4800 mg/60 kg *∗* 6.2≈504 mg/kg·day). The main active principles of Shengjing capsules are displayed in Table 1. In order to ensure the experimental quality, the experimental Shengjing capsule was dissolved in distilled water to prepare the designated Shengjing capsule concentrations.

### 2.2. Animals and Experimental Design

Twenty-five male SPF Sprague–Dawley rats (weight 120–140 g, 49–51 days old) were purchased from Guangdong Medical Laboratory Animal Center (Laboratory Animal License Number SYXK (Yue) 2013-0093). The animal room was kept under 12-hour light/dark cycles and maintained at 20 ± 2°C with 45%–65% relative humidity. The animals had free access to food and water. All the rats were randomly divided into five groups (five rats each): (1) the normal group: the rats were treated daily with distilled water (2 ml) by oral gavage; (2) the NOA group: the rats underwent an NOA modeling, which was conducted by intraperitoneally injecting busulfan 10 mg/kg on day 1 and day 21, as reported previously [20]. At 35 days after second busulfan injection, azoospermia was confirmed in these rats. The NOA rats were treated daily with distilled water (2 ml) by gavage; and (3) the three Shengjing treatment groups: the NOA rats were treated daily with a (a) high dose of Shengjing capsules (1008 mg/kg), (b) medium dose of Shengjing capsules (504 mg/kg), or (c) low dose of Shengjing capsules (252 mg/kg) by gavage. The Shengjing capsules were dissolved in distilled water (2 ml). Intragastric gavage was for 8 weeks, and the body weight of each animal was registered every week. After that, the rats were anesthetized. We obtained the specimens from the rats for tests of corresponding indexes. The rat's testes and epididymides were weighted to get their organ indexes.

All experimental protocols were subject to approval by the Institutional Animal Care and Use Committee of the First Affiliated Hospital of Guangzhou Medical University (Guangzhou, China).

### 2.3. Sperm Count

To determine the sperm morphology, the animals were sacrificed after anesthesia. Then, the cauda epididymides of all animals were quickly transferred, and epididymal fluid was observed in the polarizing microscope. Then, they were minced in 10 mL prewarmed 0.9% normal saline to incubate at 37°C for 10 minutes that allowed sperm to swim out of the lumen of the cauda epididymides for sperm characteristic analysis.

The sperm count was determined using the haemocytometer under light microscope. A cover slip was placed on the haemocytometer before a 10 *μ*l drop of caudal epididymal sperm solution was loaded under the cover slip. The haemocytometer was placed under the light microscope and viewed under ×400 magnification. The sperm count was calculated by counting 4 × 4 squares (horizontally or vertically) [21].

### 2.4. Histological Examination and Morphological Changes of Seminiferous Tubules

An abdominal incision was made, both sides of testes were dissected out, and part of them were fixed in Bouin's fixative for histological investigations and subsequently embedded in paraffin. The embedded tissues were cut into 4 *μ*m thicknesses. Then, tissue sections were deparaffinized in xylene and hydrated in descending concentrations of ethanol before hematoxylin and eosin (HE) staining. Morphological changes were observed under a microscope, for example, the morphology of convoluted tubules, the presence of vacuolization, and the death and degeneration of SSCs and spermatocytes. For histomorphometric analyses, cells were recorded in 50 random seminiferous tubules to compare the differences in average cell numbers among the five groups.

### 2.5. Quantitative Reverse Transcription-Polymerase Chain Reaction (qRT-PCR)

Expression levels of *α*6-integrin and *β*1-integrin mRNA in five groups were assayed by qRT-PCR analysis in accordance with the protocol [22]. The comparative Ct method was utilized to calculate the relative changes on the real-time PCR system. The primer sequences are summarized in Table 2.

### 2.6. Protein Extraction and Western Blot Analysis

Total protein was extracted from testicular tissue. Western blot analysis was conducted as previously published by our laboratory [22]. The membrane was incubated with Anti-Integrin *α*6 (1 : 2000 dilution; ab181551, Abcam), Anti-Integrin *β*1 (1 : 2000 dilution; ab179471, Abcam), phospho-Akt polyclonal rabbit antibody at a dilution of 1 : 2000 (ab81283, Abcam), and phosphoinositide 3-kinase (PI3K) at a dilution of 1 : 2000 (4257, CST) overnight at 4°C and incubated with anti-rabbit IgG horseradish peroxidase conjugated secondary antibodies (1 : 3000 dilution; ab136817, Abcam). The relative protein expression was normalized with GAPDH (1 : 3000 dilution; ab8245, Abcam).

### 2.7. Immunohistochemistry

Testicular tissue section slides in five groups were heated at 60°C for approximately 1 hour in a hot air oven. Then, they were deparaffinized in xylene and rehydrated using alcohol gradient. The antigen retrieval process was performed. Then, they were cooled to room temperature. The primary antibodies used were p-Akt (ab81283, Abcam) at a 1 : 200 dilution, Anti-Integrin *α*6 (1 : 200 dilution; ab181551, Abcam), and Anti-Integrin *β*1 (1 : 500 dilution; ab179471, Abcam). After stained according to the standard immunohistochemical protocol, immunoreactivity was evaluated by assessing positive cell percentages and staining intensities. The percentage scoring of immunoreactive cells was as follows: 0 (0–5%), 1 (6–25%), 2 (26–50%), 3 (51–75%), and 4 (>75%). The staining intensity was visually scored and stratified as follows: 0 (negative), 1 (weak), 2 (moderate), and 3 (strong). A final immunoreactivity score (IRS) was obtained for each case, multiplying the percentage and the intensity score.

### 2.8. Statistical Analysis

SSPS 13.0 software (SPSS, Chicago, IL, USA) was utilized to analyze the experimental data. All experiments were performed more than three times, the data of this research were described as means standard deviations (SDs), and comparisons were performed using Student's *t*-tests. The one-way ANOVA was done for comparison among different groups. *P* < 0.05 was examined statistically significant.

## 3. Results

### 3.1. Body Weights and Tissue Weights of Testis and Epididymis

The initial body weight did not differ significantly among the five groups (*P* > 0.05). After Shengjing capsule treatment, we weighted body, testicular, and epididymal weights to establish the declining trend of spermatogenesis. As shown in Table 3, testicular and epididymal weights of two sides of the rats from the NOA group reduced significantly (48.6% and 62.2%; *P*=0.0001 for both) in comparison with the normal group. In Shengjing treatment groups, the testicular and epididymal weights in the medium-dose group increased significantly (*P*=0.001 for all) compared to the NOA group. Similarly, there was a significant improvement in testicular and epididymal weights in the high-dose group compared to the NOA group (*P*=0.0001 for all). No significant differences were detected in these data between the NOA group and low-dose group (*P*=0.957 and *P*=0.293). No obvious difference in body weight was proved among the five groups. The findings presented here suggested a beneficial effect of Shengjing capsules on spermatogenesis in the NOA rats.

### 3.2. Sperm Counts and Sperm Quality

**Figure 1:**
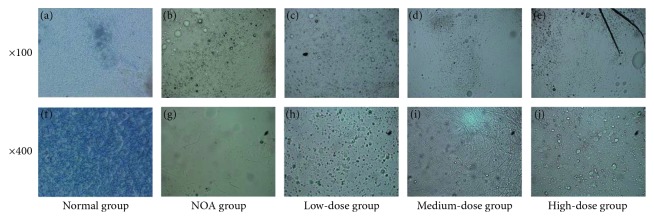
Sperm counts of five groups.

We examined sperm counts to evaluate the therapeutic efficacy of Shengjing capsules on spermatogenesis by epididymal fluid smear examination. As shown in Figure 1, under the polarizing microscope, the NOA group had less sperms, while the sperm count of the normal group was all over the horizon. There were a few sperms in the low-dose group, while the sperm counts of the medium-dose group and high-dose group have increased with different degrees. There was a similar trend, as shown in Table 4, when we quantitated the counts of sperms. The counts of sperms in the NOA group were strongly lower than those in the normal group (*P*=0.0001; Table 4; Figure 1), while the counts in the medium-dose group and high-dose group were significantly higher than those in the NOA group (*P*=0.0001; Table 4; Figure 1). Sperms in the low-dose group also increased when compared with the NOA group (*P*=0.034; Table 4; Figure 1).

### 3.3. Histological Morphology of Spermatogenesis

**Figure 2:**
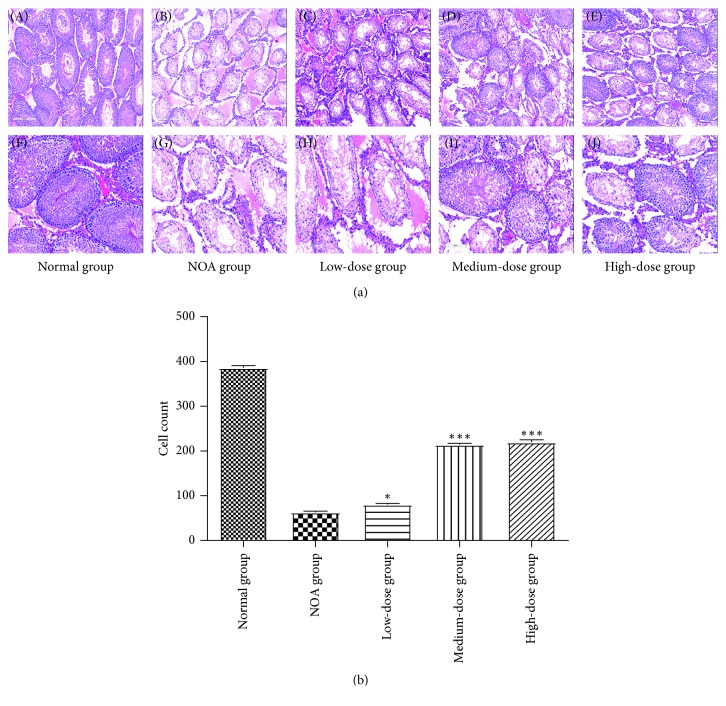
Morphology of seminiferous tubules examined with HE staining. (a) HE-stained sections of testicular tissues from each group. Lower panel: a higher magnification of the upper panel. (b) Average cell counts in 50 seminiferous tubules in each group. ^*∗*^*P* < 0.05 versus the NOA group; ^*∗∗∗*^*P* < 0.0001 versus the NOA group.

We analyzed the histology of the seminiferous tubules after Shengjing capsule treatment. In the normal group, layers of germ cells, spermatogonia, and spermatocytes were clearly defined, and sperm cells were observed in the lumen of the tubules. However, after double dose of busulfan, spermatogonia and spermatocytes appeared vacuolated, resulting in disturbed seminiferous tubular layers and disappearance of spermatogonia, spermatocytes, and spermatids. In the NOA group, they were manifested by atrophy and sparse arrangement of contorted seminiferous tubules. Almost all of the seminiferous tubules across the section were vacuolated with a thin layer of basal compartment containing Sertoli cells and some SSCs. In the low-dose group, part of seminiferous tubules across the section had a thin layer of SSCs and spermatocytes without sperm cells. In medium- and high-dose groups, there were more layers of SSCs and spermatocytes, part of which had intracavitary sperms. The incidence of spermatogenesis hypofunction gradually decreased as the dosage of Shengjing capsule increased, which meant that the therapeutic efficacy and dosage were closely interrelated. Fifty seminiferous tubules in the medium-dose group and high-dose group were counted, and there were 214.40 ± 6.69 cells and 219.60 ± 12.64 cells after increase, respectively, compared to 63.20 ± 4.97 cells in the NOA group (*P*=0.0001 for both; Figure 2).

### 3.4. Expression of Integrin *α*6/*β*1

**Figure 3:**
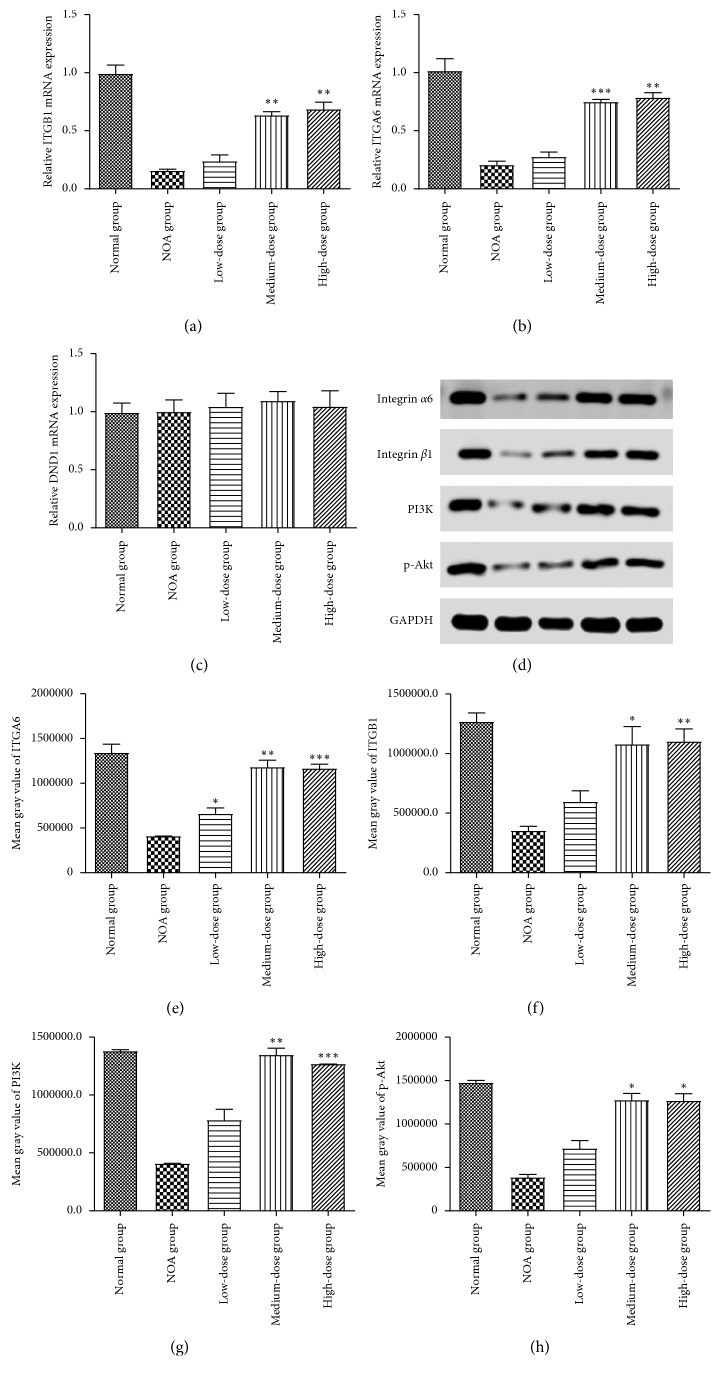
(a, b) Expression levels of *α*6-integrin and *β*1-integrin mRNA are presented. (c) Expression levels of DND1 mRNA are presented. (d) Western blot analysis showing the protein expression. (e–h) Densitometry and statistical analysis of *α*6/*β*1-integrin and PI3K and p-Akt proteins (ratio to GAPDH). qRT-PCR and western blot were conducted three times, and the results are expressed as means ± standard deviations (SDs). ^*∗*^*P* < 0.05 versus the NOA group; ^*∗∗*^*P* < 0.01 versus the NOA group; ^*∗∗∗*^*P* < 0.0001 versus the NOA group.

In this study, quantitative RT-PCR and western blot results showed that *α*6-integrin and *β*1-integrin were significantly downregulated in the NOA group compared with the normal group. This finding reflected the loss of spermatozoa and fertility. The PCR results were verified by western blot. The expression of integrin *α*6/*β*1 declined significantly in the NOA group compared with the normal group (Figure 3). In Shengjing treatment group, the integrin *α*6/*β*1 expression significantly increased in the medium-dose and high-dose groups compared with the NOA group, while there was no statistical significance in the low-dose group (Figures 3(a), 3(b), 3(e), and 3(f); *P* < 0.01 for all). Furthermore, densitometric analysis of the western blotting bands revealed that values of *α*6-integrin were 0.40, 0.59, 1.23, and 1.27 for NOA and low-, medium-, and high-dose groups, respectively (Figure 3(e)). The expression values of *β*1-integrin were 0.23, 0.53, 1.02, and 1.11 for NOA and low-, medium-, and high-dose groups, respectively (Figure 3(f)). Compared with the NOA group, the expression level of *α*6-integrin increased by 2.08-fold and of *β*1-integrin by 1.92-fold in the medium-dose group (*P* < 0.01 for both; Figure 3). The results of PCR demonstrated that no significant difference was found in the expression level of DND1 among the five groups (Figure 3(c); *P* > 0.05 for all).

**Figure 4:**
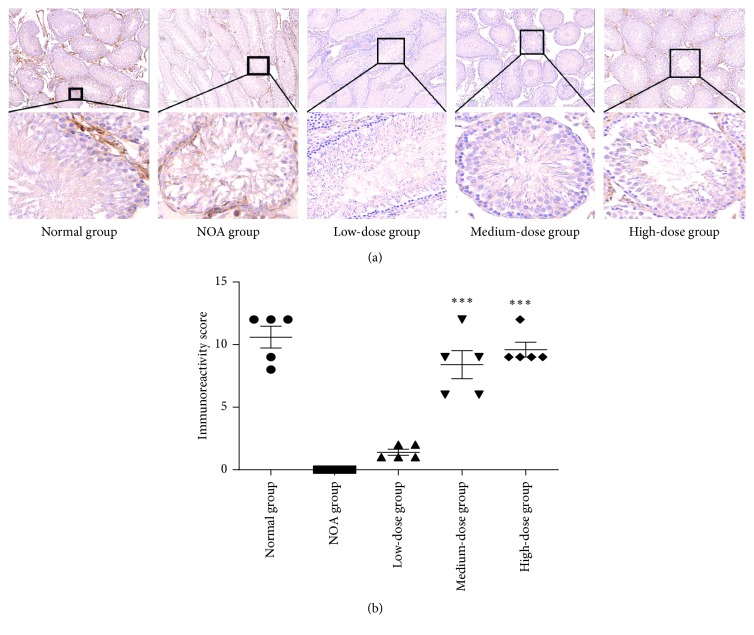
Immunohistochemical analysis of the *α*6-integrin expression in the rat testis. Lower panel: a higher magnification of the upper panel. ^*∗∗∗*^*P* < 0.0001 versus the NOA group.

**Figure 5:**
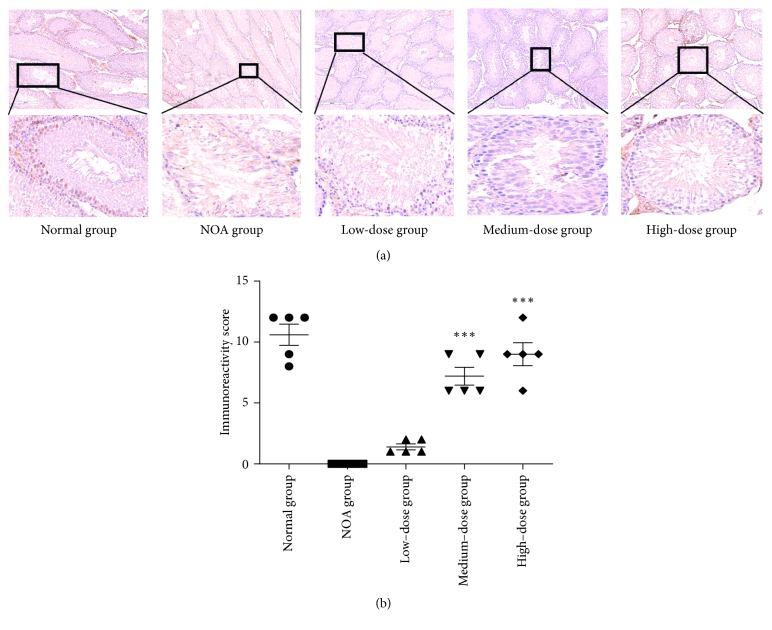
Immunohistochemical analysis of the *β*1-integrin expression in the rat testis. Lower panel: a higher magnification of the upper panel. ^*∗∗∗*^*P* < 0.0001 versus the NOA group.

Immunohistochemistry was performed to assess the integrin *α*6/*β*1 expression in the SSCs (Figures 4 and 5). The mean staining intensity of *α*6-integrin was significantly lower in the NOA group than that in the normal group (0.00 ± 0.00 versus 10.60 ± 1.95; *P*=0.0001; Figure 4). The staining intensity was significantly stronger in medium-dose and high-dose groups than that in the NOA group (*P*=0.002 and *P*=0.0001). Similar results were presented for *β*1-integrin (Figure 5).

### 3.5. Shengjing Capsule Upregulated PI3K/AKT Signaling Pathway

The PI3K/AKT pathway is critical to cell proliferation, cell growth, differentiation, and spermatogenesis [6, 7, 23–25]. However, the mechanism remains unknown. The correlation between PI3K/AKT and integrin *α*6/*β*1 in spermatogenesis has been unexplored yet.

**Figure 6:**
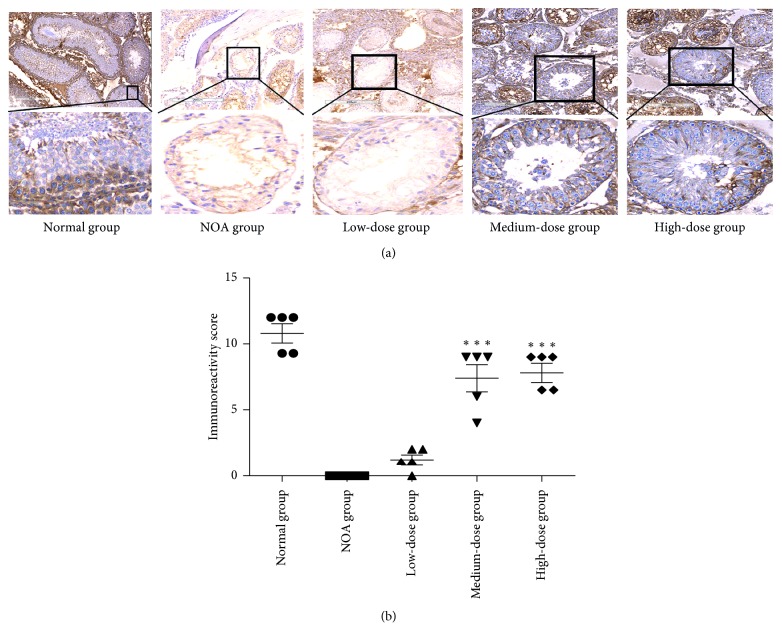
Immunohistochemical analysis of the p-Akt expression in the rat testis. Lower panel: a higher magnification of the upper panel. ^*∗∗∗*^*P* < 0.0001 versus the NOA group.

To detect the expressions of PI3K and Akt in testes, we first confirmed the expression of phosphorylated protein kinase B (p-Akt) by immunohistochemistry. After Shengjing capsule treatment, an evident expression of p-Akt can be observed in the cells near the basal lamina of seminiferous tubules in rat testes, where SSCs are located (Figure 6). The brims of SSCs close to the basal lamina were evidently stained in medium- and high-dose groups compared with the normal group. For the SSCs of seminiferous tubules in the NOA group, they were scarcely stained. Then, we detected the expressions of PI3K and p-Akt through western blotting (Figures 3(d), 3(g), and 3(h)). PI3K and p-Akt expressions increased in medium-dose and high-dose groups, but they scarcely increased in the low-dose group. In quantitative analysis, compared with each respective normal group, the densitometry values of the immunoreactive bands concerning PI3K were 0.43, 0.88, 1.41, and 1.28 for NOA and low-, medium-, and high-dose groups, respectively (Figure 3(g)). Values of p-Akt were 0.44, 0.54, 1.12, and 1.12 for NOA and low-, medium-, and high-dose groups, respectively (Figure 3(h)). p-Akt expression levels also increased significantly in medium- and high-dose groups when compared with the NOA group (*P*=0.0135 and *P*=0.0206).

## 4. Discussion

Patients with azoospermia can achieve pregnancy by extracting sperm from the testicles through assisted reproductive technology [26]. For NOA patients, TESE and testicular sperm aspiration (TESA) are the most common methods for sperm extraction [27]. However, the possibility of finding sperm cells is only about 50% in NOA patients. The disadvantage of this invasive procedure is that testicular tissue may transiently or permanently affect androgen production [28]. Therefore, finding effective and feasible treatments can be an important adjuvant treatment for NOA patients. Traditional Chinese herbal medicines have a history of thousands of years and have the potential to improve sperm production in NOA patients. Shengjing capsules have the potential to improve the sperm production ability of NOA patients. As a noninvasive treatment, the Shengjing capsule is easy to operate and does not cause testicular damage.

In this study, we used HE staining and semen smear to find that the number of spermatogenic epithelial cells was higher in the Shengjing treatment group, and the sperm count was higher than that in the NOA rat group. It showed the disturbance in the seminiferous tubular layers and the disappearance of spermatocytes and spermatids in the NOA group. Almost all the seminiferous tubules across the section were vacuolated with a thin layer of the basal compartment containing Sertoli cells and some SSCs in HE staining. After 8 weeks of Shengjing capsule treatment, the count of the spermatogenic cells, the germ cells, and even the sperms in the medium-dose and high-dose groups was significantly higher than that in the NOA group. These results indicated that the Shengjing capsule could repair the damaged seminiferous epithelium and protect spermatogenesis.

The spermatogenic function of azoospermia rats was improved in the medium- and high-dose spermatogenic capsule groups. However, the spermatogenic function of rats did not increase with the increasing dose of the Shengjing capsule. This is consistent with the clinical use of Shengjing capsules to improve the semen quality of azoospermia patients. These results suggest that Shengjing capsules may have similar effects on improving semen quality in NOA but require more multicenter studies with larger samples.

Spermatogenesis is a complex process that originates with and depends on SSCs. Spermatogonial self-renewal is a critical prerequisite for the maintenance of normal sperm counts. And the disruption in their function has dire consequences on fertility. These primitive cells reside on the basement membrane of the seminiferous tubule and slowly proliferate to provide (a) additional stem cells by self-renewal and (b) progeny cells that undergo significant amplification during the differentiation process to spermatozoa [29]. A single SSC in a rat is capable of producing more than 4,000 sperm, most of which disappear in the normal process by programmed cell death [30].

Stem cells have been reported as a star therapeutic target in a number of cases of busulfan causing NOA model literatures [31]. SSC has a unique self-renewal capacity to maintain the basic number of cell pools from which progenitor cells that are committed to the terminal differentiation pathway are produced [32]. Integrin *α*6/*β*1 is an important biomarker of SSCs. These effects make it possible for SSCs to support homeostasis and provide sperm regeneration [33].

In this study, we observe that Shengjing capsules significantly improve the spermatogenesis in the NOA rats. To elucidate the underlying mechanisms, we detect the expression of the specific biomarker of *α*6-integrin and *β*1-integrin for SSCs in testicular tissue. We find that the integrin *α*6/*β*1 expression is barely expressed in the NOA group. And in medium-dose and high-dose groups, the expression levels of integrin *α*6/*β*1 significantly increased compared with the NOA rat group. It means that a significant increase is shown in the numbers of SSCs after Shengjing capsule treatment, though the improvement does not increase with the dose. Since the vertebrate-conserved RNA-binding protein DND1 is necessary for the survival of SSCs, we further examine the DND1 expression. Taken together, these findings reveal that Shengjing capsules might activate SSCs via upregulating integrin *α*6/*β*1.

The PI3K/AKT pathway might play an impotent role in male fertility [34]. Male mice with a homozygous mutation of the PI3K pathway are sterile because of a block in spermatogenesis, with initially decreased proliferation and subsequent extensive apoptosis occurring at the SSC level. The findings of De Miguel et al. [35] suggested an important role of AKT/mTOR in mediating primordial germ cell growth, spermatogonial proliferation, and spermatogenesis. In this study, the expression levels of PI3K and p-Akt have a similar trend of integrin *α*6/*β*1. Western blotting analysis demonstrated that PI3K and p-Akt expressions apparently enhanced in medium- and high-dose Shengjing capsule groups compared with the normal group. Immunohistochemistry analysis corroborated these results, showing that expressions of PI3K, p-Akt, and integrin *α*6/*β*1 were significantly higher in medium-dose and high-dose Shengjing capsule groups than in the NOA group. PI3K/AKT may play an important role in the improvement of azoospermia when using Shengjing capsules for upregulating integrin *α*6/*β*1.

The Shengjing capsule comprises 19 ingredients, some of which are used to improve testicular function. For instance, the *Panax ginseng* extract could enhance testicular function by elevating GPx and GST activity, thus resulting in increased glutathione, which prevented LPO in the testis [36]. Kopalli et al. [37] also reported that the *Panax ginseng* extract attenuates H_2_O_2_-induced spermatocyte oxidative stress in mouse spermatocytes and regulates the expression of antioxidant-related genes, spermatogenesis-related proteins, and sex hormone receptors in aged rats. Ginseng may be a potential natural drug used to protect from or treat stress-induced male infertility. Zhang et al. [38] reported that *Lycium barbarum* could improve sperm density, sperm movement, and the rate of normal sperm morphology. *Lycium barbarum* could also reduce cadmium-induced testicular damage by increasing antioxidant enzyme activity and reducing oxidative stress. It may be a potential adjunct to testicular toxicity. Taken together with the findings presented here, Shengjing capsules, as a Chinese herbal medicine, may activate SSCs into the process of spermatogenesis by upregulating the expression of integrin *α*6/*β*1 through the PI3K/AKT signal pathway in the NOA rats.

### 4.1. Limitations

When evaluating spermatogenic function of rats, only the changes of sperm number were evaluated. No other parameters of sperm quality were compared, such as sperm motility and deformity rate. This is due to the lack of professional evaluation of the rat sperm instrument and cannot carry out effective assessment of rat semen quality. Meanwhile, sperm count is one of the indicators of semen quality, and the increase in the sperm count partly reflects the restoration of spermatogenesis in mice. We did not interbreed rats among the treatment groups to evaluate the fertility achieved by the Shengjing capsules in rats. The restoration of spermatogenesis could theoretically lead to the restoration of fertility in mice. More relevant research is needed to clarify the mechanism of Shengjing capsules in improving spermatogenic function in patients with NOA.

### 4.2. Strengths

Traditional Chinese medicine for azoospermia enriches the present-day therapies in the field of sexual medicine. To our knowledge, this is the first study to evaluate the therapeutic effect of Shengjing capsules on NOA. We elaborate a possible potential mechanism and provide a new direction for the treatment of NOA, and further research is needed to clarify the possible mechanism of action. It provides evidence for the clinical treatment of NOA patients using Shengjing capsules.

## 5. Conclusion

This study firstly shows that the Shengjing capsule significantly improves the spermatogenesis in the NOA rats. The underlying mechanisms might involve activating SSCs by upregulating the integrin *α*6/*β*1 expression via the PI3K/AKT pathway. Our results suggest Shengjing capsules as a promising therapeutic medicine for NOA.

## Figures and Tables

**Table 1:** Main active principles of Shengjing capsules.

Formula	Percentage
Lu Rong (Pilose Antler)	10.53
Gou Qi Zi (*Lycium barbarum*)	10.53
Ren Shen (*Panax ginseng*)	10.53
Dong Chong Xia Cao (*Cordyceps sinensis*)	10.53
Yin Yang Huo (*Epimedium* herb)	10.53
Sha Wan Zi (*Astragalus complanatus*)	5.26
Tu Si Zi (Semen Cuscutae)	5.26
Huang Jing (Rhizoma Polygonati)	5.26
He Shou Wu (*Polygonum multiflorum*)	5.26
Sang Shen (mulberry)	2.63
Bu Gu Zhi (*Psoralea corylifolia*)	2.63
Gu Sui Bu (Rhizoma Drynariae)	2.63
Xian Mao (*Curculigo orchioides*)	2.64
Jin Ying Zi (*Rosa laevigata* Michx.)	2.63
Fu Pen Zi (*Rubus chingii*)	2.63
Du Zhong (*Eucommia ulmoides*)	2.63
Da Xue Teng (*Sargentodoxa cuneata*)	2.63
Ma Bian Cao (*Verbena officinalis*)	2.63
Yin Xing Ye (*Ginkgo biloba* leaves)	2.63

**Table 2:** Primers used for detection of the mRNA expression by RT-PCR.

Gene	Sequences	
	Forward	Reverse
GAPDH	AGGTCGGTGTGAACGGATTTG	GGGGTCGTTGATGGCAACA
ITGA6	GTTGTGCTTGCTCTACCTGTCC	GCGAGCGAGAAGCCGAAGAG
ITGB1	GAATGGAGTGAATGGGACAGGAG	CAGATGAACTGAAGGACCACCTC
DND1	CTCCCTCTTAGCTTGAACCGACG	ACTCCACAGCCACCTGCTCT

ITGA6 = *α*6-integrin; ITGB1 = *β*1-integrin.

**Table 3:** Organ weight information in different groups.

(g)	Normal group	NOA group	Low-dose group	Medium-dose group	High-dose group
Body weight	515.56 ± 1.77	514.22 ± 13.79	514.43 ± 12.75	513.91 ± 27.61	515.64 ± 27.79
Weights of testis	2.90 ± 0.35	1.49 ± 0.14^*∗*^	1.60 ± 0.17	2.20 ± 0.40^*∗*^	2.34 ± 0.22^*∗*^
Weights of epididymis	1.11 ± 0.07	0.42 ± 0.03^*∗∗*^	0.44 ± 0.37	0.80 ± 0.16^*∗∗*^	0.82 ± 0.35^*∗∗*^

Two sides of testes were weighted together as well as the epididymides (five rats per group). ^*∗*^*P* < 0.05; ^*∗∗*^*P* < 0.01 (one-way ANOVA for comparison among different groups).

**Table 4:** Sperm analysis information in different groups.

Sperm analysis	Normal group (10^6^/mL)	NOA group (10^2^/mL)	Low-dose group (10^2^/mL)	Medium-dose group (10^6^/mL)	High-dose group (10^6^/mL)
Sperm count	63.76 ± 3.22	5.49 ± 1.34	14.18 ± 4.17^*∗*^	5.71 ± 3.81^*∗∗∗*^	7.28 ± 5.32^*∗∗∗*^

There were five rats per group. ^*∗*^*P* < 0.05; ^*∗∗∗*^*P* < 0.0001 (one-way ANOVA for comparison among different groups).

## Data Availability

The data used to support the findings of this study are available from the corresponding author upon request.
